# 

**Published:** 1950-10

**Authors:** 


					kifCOMTAl ' ?1DEX1'
I PUBLISHED UNDER THE AUSPICES OF THE BRISTOL MEDICO-CHIRURGICAL SOCIETY
THE BRISTOL
MEDICO - CHIRURGICAL
JOURNAL
A Journal of the Medical Sciences for the
WEST OF ENGLAND
AND SOUTH WALES
Editor:
E. WATSON-WILLIAMS, M.C., M.D., Ch.M., F.R.C.S.
with whom are associated
G. E. F. SUTTON, M.C., M.D., F.R.C.P.
J. H. MIDDLEMISS, M.D., D.M.R.D., F.F.R.
N. S. CRAIG, M.C., M.D., Assistant Editor.
Local Correspondents :
BATEMAN, O.B.E., F.R.C.S.
/' A,r\m ' ExeterV-L. N. JACKSON, M.C., D.M.
* p* r GO^ucester?RaF. JARRETT, M.B., M.R.C.P.
' ' *""'Sport?J. L.Vp. WILLIAMS, M.B., F.R.C.S.
PlR.^HoFT, D.M., M.R.C.P. Taunton?Dr. M. McALLEY, D. M.R.
Torquay?Dr. A^ROBINSON THOMAS, D.M.R.E.
\ TRURO-j^^yj^^KD^'.R.La Yeovil?J. A. VERE NICOLL, F.R.C.S.
BRISTOL I
J. W. ARROWSMITH LTD., QUAY STREET AND SMALL STREET
I^ol. LXVn. No. 244. Price: FOUR SHILLINGS net.
OCTOBER * 1950
}?!!!!
BBBH

				

